# Investigation of biomarkers alterations after an acute tissue trauma in human trapezius muscle, using microdialysis

**DOI:** 10.1038/s41598-018-21185-4

**Published:** 2018-02-14

**Authors:** Line Bay Sørensen, Parisa Gazerani, Karin Wåhlén, Nazdar Ghafouri, Björn Gerdle, Bijar Ghafouri

**Affiliations:** 1 Center for Neuroplasticity and Pain (CNAP), SMI®, Department of Health Science and Technology, School of Medicine and Health, Aalborg University, Aalborg, Denmark; 2 Pain and Rehabilitation Centre, and Department of Medical and Health Sciences, Linköping University, Linköping, Sweden

## Abstract

Alterations in muscle milieu are suggested as important activity of peripheral drive in patients with chronic musculoskeletal pain (CMP). Microdialysis (MD) has been used in monitoring altered metabolic response pattern in muscles. However, the insertion of MD probe causes a local tissue trauma. Whether and how metabolites in trapezius muscle are affected by acute tissue trauma is unknown. Hence, this study investigated the metabolic response and nociceptive reaction of the tissue following MD probe insertion in patients with CMP and healthy individuals. Fifty-nine patients and forty pain-free volunteers were recruited. Pressure pain thresholds (PPTs) were obtained at the trapezius and tibialis muscles. Pain questionnaires determined the levels of pain related aspects. MD (20 kDa cut-off) was performed in the trapezius and samples were collected within 40 min. Interstitial concentration of the metabolites was analyzed by a two-way-mixed-ANOVA. The metabolic response pattern changed over time and alterations in the level of metabolites could be seen in both CMP and healthy controls. Pain questionnaires and pain intensities manifested clinical aspects of pain closely to what CMP patients describe. Analyzing metabolites due to acute tissue trauma by aid of MD may be a useful model to investigate altered metabolic response effect in CMP.

## Introduction

Chronic Musculoskeletal Pain (CMP) is referred to a variety of different pain conditions affecting deep tissue structures in human body. The clinical description is often presented by diffuse pain localization, referrals, and deep tissue hyperalgesia or allodynia^1^. CMP is characterized as either regional, localized or widespread pain and severe states affect approximately 20% of the adult population in Europe with higher prevalence in women^2,3^. The majority of this population experience negative consequences related to the chronic pain problem affecting their socio-economic status and work-related activities. Hence, a reduced quality of life is reported^3^. Often the pain is a symptom driven by sensory inflammatory disturbances or tissue injury as a result of repetitive strain or work-related conditions^4^. On consensus, a complex network including biochemical pathways and psychological factors play a fundamental role. However, potential underlying mechanisms maintaining the ongoing pain are not well achieved but alteration in muscle milieu has been suggested as an important activity of peripheral drive in patients with CMP^5–7^. The Microdialysis (MD) technique has been used to provide important information on release of pain related substances and metabolic alterations in the trapezius muscle of chronic pain patients^8–10^. Higher interstitial concentrations of lactate and pyruvate have been found in patients with fibromyalgia and localized chronic myalgia^10^. In addition, increased levels of glutamate and lactate in muscles have been identified in patients with chronic widespread pain compared to healthy controls^8^. Since insertion of a MD probe causes local tissue trauma, a recovery period has been introduced before sampling in prior studies investigating muscle characterization and metabolism^11^ as the release of substances from the injured tissue may influence the findings^12^. A trauma phase period within 2 hours is normally considered to allow tissue normalization^12^. It is unclear however, if such inflammatory response would act on nociceptors and sensitize those^13^. This is in particular important feature as sensitization of nociceptors may last longer than the actual release time when it is initiated, and if such peripheral activity drive continues or become recurrent, it may lead to central sensitization that can be manifested as hypersensitivity in chronic pain patients. Whether metabolic alterations occur during this acute trauma phase are currently unknown. Initial steps have been taken by Gerdle *et al*.^14^ comparing levels of metabolites in the muscle collected during trauma phase of MD in women with neck-shoulder pain (CNSP), chronic widespread pain (CWP) and healthy controls. Although results indicate that different type of chronic pain conditions show different metabolic response pattern compared to healthy controls, questions remain whether this could feature a distinct response based on the type of chronic pain condition.

Therefore, this study aimed at investigating the metabolic response pattern and nociceptive reaction of the tissue in the trapezius muscle of patients with CMP and healthy controls during 40 min of acute trauma introduced by a MD probe. In addition, we examined if any differences in metabolic concentrations would be related to sex and specific sub-population of patients in CMP.

## Results

### Patients with chronic musculoskeletal pain (CMP) and healthy controls (CON)

All participants completed the study without any detection or reports of safety issues. Four patients and one control subject were excluded from data analysis of the questionnaires due to lack of response. No significant differences in background data were seen between CMP (51 females and 8 males) and CON (30 females and 10 males) except for the BMI (P = 0.044, Table 1). Furthermore, significant differences regarding depressive and anxiety symptoms as well as catastrophizing impact were found between the two groups (P ≤ 0.05, Table 1). However, at group level, both groups had score values below 11 on both subscales of the Hospital Anxiety and Depression Scale (HADS, i.e. no potential case with respect to anxiety or depression).

**Table 1:** Demographic and clinical data from patients with musculoskeletal pain vs. healthy controls and subgroup comparisons.

Variables	CMP (n = 59)	CON (n = 40)	Statistic (P-value)	Fcmp (n = 51)	Fcon (n = 30)	Statistic (P-value)	Mcmp (n = 8)	Mcon (n = 10)	Statistic (P-value)	CON (n = 40)	Reg (n = 35)	Gen (n = 24)	Statistic (P-value)
*Age (years)*	43.7 (10.6)	39.6 (8.9)	0.051	43.1 (10.0)	40.5 (9.4)	0.244	47.3 (14.1)	37.1 (7.6)	0.068	39.6 (8.9)	43.3 (10.7)	44.3 (10.7)	0.139
*Weight (kg)*	74.9 (14.9)	73.3 (12.3)	0.562	72.3 (13.8)	70.3 (11.9)	0.515	91.9 (11.1)	82.2 (8.7)	0.054	73.3 (12.3)	70.8 (11.8)	85.0 (16.98)	0.017* ^**†**^
*Height (cm)*	166.3 (21.5)	172.8 (8.5)	0.085	164.2 (22.1)	169.6 (6.9)	0.198	181.6 (4.2)	182.3 (5.0)	0.765	172.7 (8.5)	168.6 (8.5)	163.5 (32.2)	0.125
*BMI (Kg/m* ^*2*^ *)*	26.2 (4.4)	24.5 (3.3)	0.044*	25.9 (4.5)	24.4 (3.5)	0.121	27.9 (3.4)	24.8 (2.8)	0.051	24.5 (3.3)	24.8 (3.3)	28.1 (5.1)	0.001* ^**†**^
**Questionnaire**	**CMP (n = 58)**	**CON (n = 40)**	**Statistic (P-value)**	**Fcmp (n = 50)**	**Fcon (n = 30)**	**Statistic (P-value)**	**Mcmp (n = 8)**	**Mcon (n = 10)**	**Statistic (P-value)**	**CON (n = 40)**	**Reg (n = 35)**	**Gen (n = 23)**	**Statistic (P-value)**
*HADS-D*	4.1 (4.6)	1.6 (2.6)	0.000*	5.4 (3.8.)	1.4 (2.1)	0.000*	7.4 (5.8)	2.2 (2.3)	0.020*	1.56 (2.5)	5.0 (3.7)	6.7 (4.5)	0.000*^†^
*HADS-A*	6.8 (4.6)	2.9 (3.0)	0.000*	6.7 (3.8)	2.5 (2.9)	0.000*	7.6 (8.1)	4.1 (8.1)	0.227	2.9 (3.0)	6.2 (3.9)	7.7 (5.4)	0.000*^†^
	**CMP (n = 56)**	**CON (n = 39)**		**Fcmp (n = 48)**	**Fcon (n = 29)**		**Mcmp (n = 8)**	**Mcon (n = 10)**		**CON (n = 39)**	**Reg (n = 33)**	**Gen (n = 23)**	
*PCS*	14.4 (8.5)	6.5 (5.9)	0.000*	14.7 (8.9)	6.5 (6.5)	0.000*	13.1 (6.1)	6.6 (4.2)	0.019*	6.5 (5.9)	15.5 (8.4)	12.9 (8.5)	0.000* ^**†**^

Values are presented as mean and (SD). *Denotes significant difference (P < 0.05) between the groups, One-Way ANOVA. ^†^Bonferroni; weight: significant difference between Reg vs. Gen (P = 0.016). BMI: significant difference between CON vs. Gen (P = 0.001) and Reg vs. Gen (P = 0.006). HADS-D, HADS-A, and PCS: significant difference between CON vs. Reg and CON vs. Gen (P ≤ 0.05). Abbreviations: CON = controls, CMP = Chronic Musculoskeletal Pain, F_cmp_ = Females with musculoskeletal Pain, F_con_ = Female controls, M_cmp_ = Males with Musculoskeletal Pain, M_con_ = Male controls, Reg = Patients with regional pain, Gen = Patient with generalized pain, HADS-D = Hospital Anxiety and Depression Scale: subscale Depression, HADS-A: subscale Anxiety, PCS = Pain Catastrophizing Scale, n = number of included subjects.

### Pressure pain thresholds (PPTs) and pain intensities

**Figure 1:**
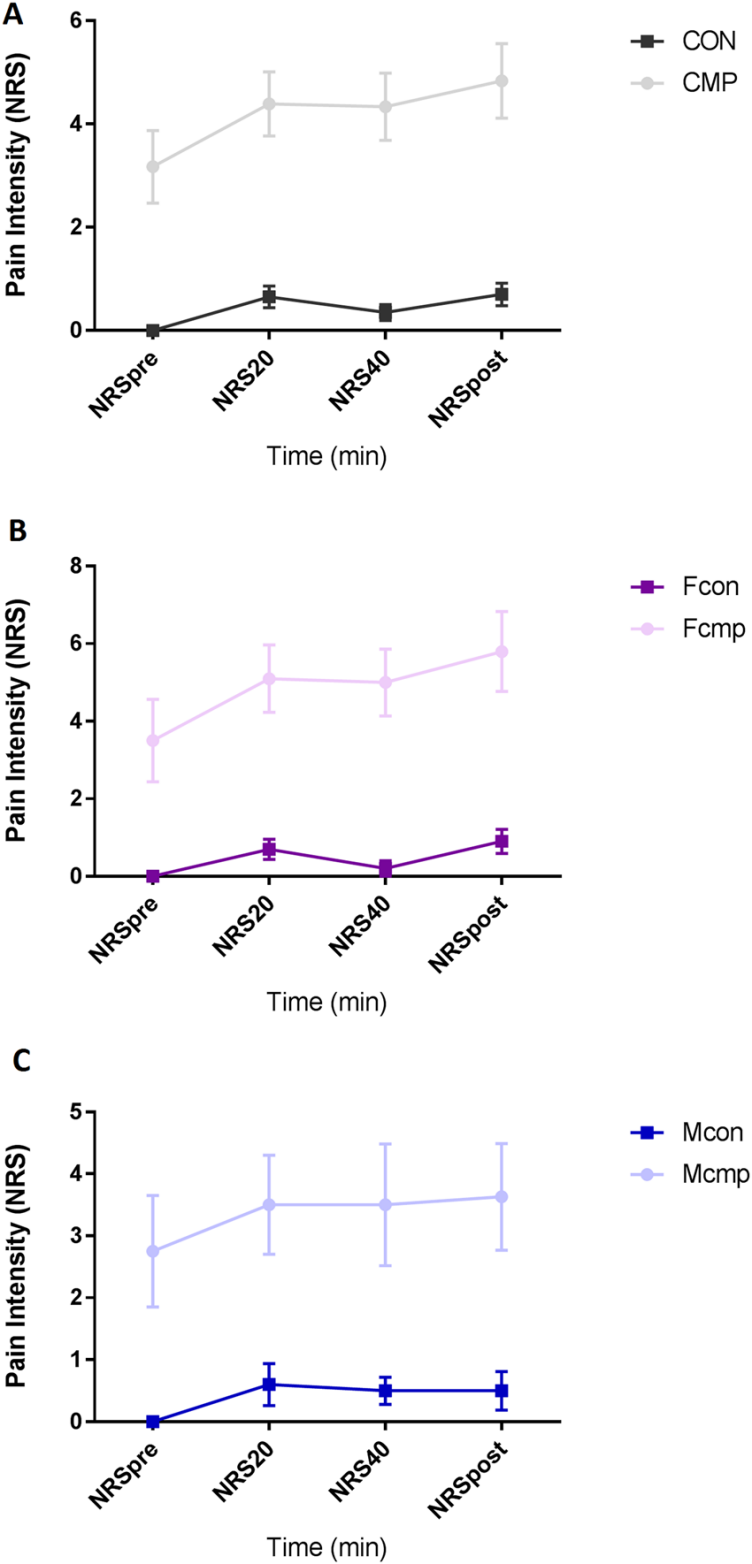
Pain intensity rating (mean and SEM) at the different time points of MD. (**A**) Shows the rating between healthy controls (CON) and patients with chronic musculoskeletal pain (CMP). (**B**) Shows the ratings between female subgroups. (**C**) Shows the ratings between the male subgroups. Overall, the patient groups show higher pain intensities throughout the MD (P < 0.05). Abbreviation: NRS = numeric rating scale (1–10), NRSpre = pain rating before MD, NRS20 = pain rating within 20 min of MD, NRS40 = pain rating within 40 min, NRSpost = pain rating after MD, CON = healthy controls, Fcmp = female patients, Fcon = female controls, Mcmp = male patients, Mcon = male controls.

Comparing PPTs over the trapezius and tibialis anterior muscles, lower PPTs were found in CMP compared to CON (Table 2). One control and four patients were excluded from data analysis due to missing values. Pain intensities were higher in CMP compared to CON at all 4 time points of evaluation during the MD (P ≤ 0.05, Fig. 1A).

**Table 2:** Pressure pain thresholds (PPTs) of trapezius and tibialis anterior muscles in patients with musculoskeletal pain vs. healthy controls and subgroup comparisons.

Variables	CMP (n = 54)	CON (n = 39)	Statistic (P-value)	Fcmp (n = 47)	Fcon (n = 29)	Statistic (P-value)	Mcmp (n = 7)	Mcon (n = 10)	Statistic (P-value)	CON (n = 39)	Reg (n = 35)	Gen (n = 19)	Statistic (P-value)
*PPT (kPa) Trapezius right*	245.7 (126.0)	456.2 (162.4)	0.000*	238.4 (122.7)	438.8 (159.9)	0.000*	294.8 (146.8)	506.8 (167.1)	0.016*	456.2 (162.4)	259.4 (117.7)	220.8 (139.9)	0.000* ^**†**^
*PPT (kPa) Trapezius left*	248.3 (146.8)	459.1 (162.6)	0.000*	244.3 (147.2)	448.3 (166.9)	0.000*	275.3 (152.1)	490.5 (152.9)	0.012*	459.1 (162.6)	273.7 (149.7)	201.4 (132.4)	0.000* ^**†**^
*PPT (kPa) Tibialis ant*.	355.6 (167.5)	540.0 (111.8)	0.000*	339.9 (164.4)	536.0 (99.3)	0.000*	460.4 (160.8)	551.2 (147.1)	0.247	540. (111.9)	384.5 (159.0)	302.3 (173.9)	0.000* ^**†**^

Values are presented as mean and (SD). *Denotes significant difference (P ≤ 0.05) between groups, One-Way ANOVA. ^**†**^Bonferroni; PPT right: significant difference (P ≤ 0.05) between CON vs. Reg and CON vs. Gen. PPT left: significant difference (P ≤ 0.05) between CON vs. Reg and CON vs. Gen. PPT tibialis: significant difference (P ≤ 0.05) between CON vs. Reg and CON vs. Gen. Abbreviations: CON = controls, CMP = Chronic Musculoskeletal Pain, F_cmp_ = Females with musculoskeletal Pain, F_con_ = Female controls, M_cmp_ = Males with Musculoskeletal Pain, M_con_ = Male controls, Reg = Patients with regional pain, Gen = Patient with generalized pain, kPa = kilopascal, n = number of included subjects.

### Interstitial concentration of the metabolites and local blood flow

**Figure 2:**
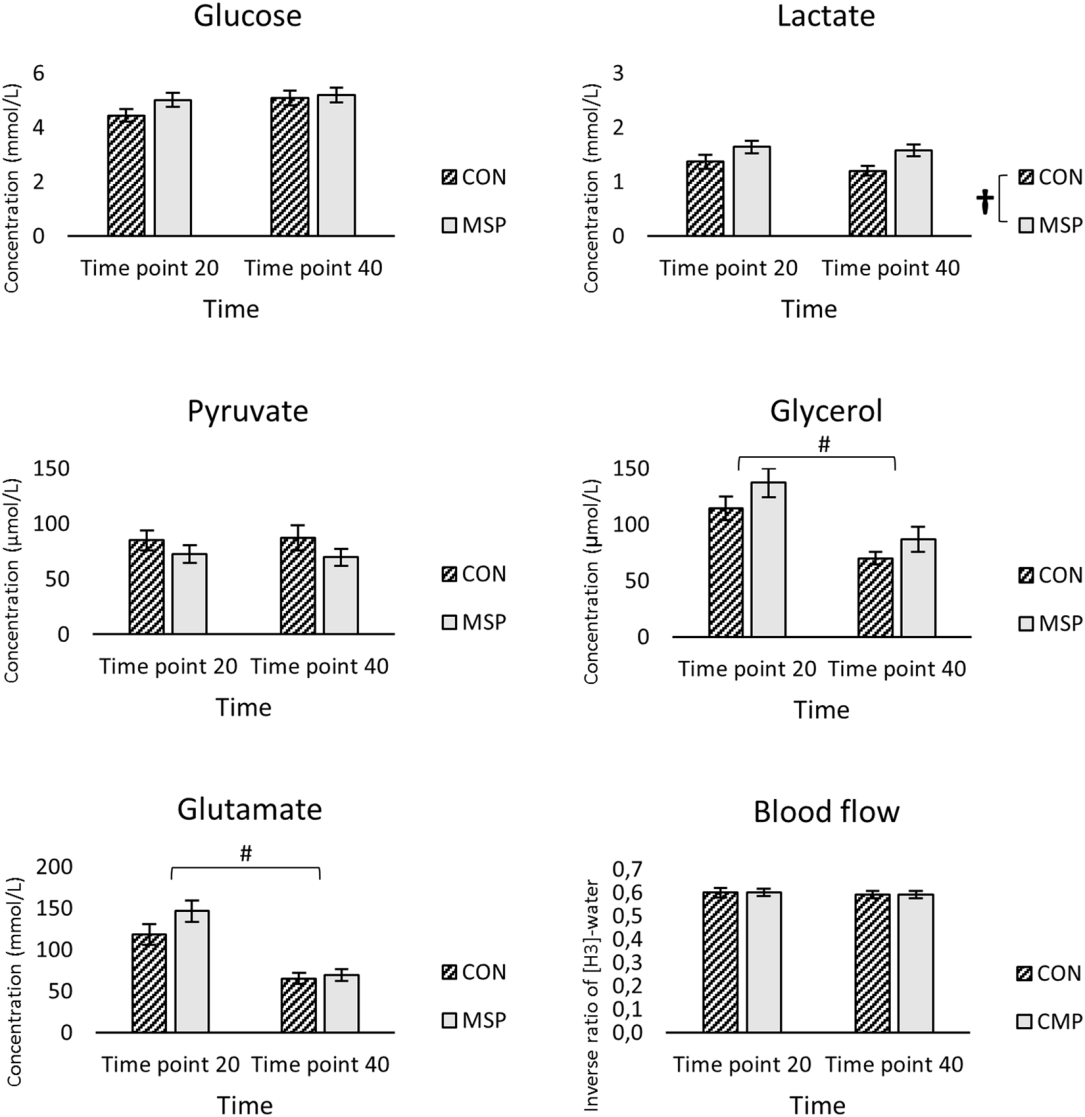
Interstitial concentrations (mean and SEM) of muscle metabolites; Glucose, Lactate, Pyruvate, Glycerol, Glutamate, and Nutritive blood flow (inverse ratio of [^3^H]-water) collected during microdialysis at two time points of 20 min and 40 min in patients with musculoskeletal pain (CMP) and healthy controls (CON). (*P ≤ 0.05) denotes significant interaction between time*group, (^#^P ≤ 0.05) denotes difference in time. (^†^P ≤ 0.05) denotes significant difference in group. Abbreviations: CMP = patients with musculoskeletal pain, CON = healthy controls. Mmol/L = millimol per. Liter, µmol/L = micromol per. Liter.

There was no interaction between the groups and time on the concentration of the metabolites (P ≥ 0.05, Fig. 2). A decrease of 47.55 (30.09–64.99) mmol/L and 65.14 (47.92–82.35) µmol/L was seen after 40 min (P ≤ 0.000) in the concentration of glycerol and glutamate respectively. At group level, CMP had 0.329 (0.05–0.61) mmol/L higher concentration of lactate than the control group (P = 0.024, Fig. 2). Local interstitial nutritive blood flow remained unchanged in both CMP and CON throughout the collecting time of MD (P = 0.95).

### Female patients (F_cmp_) and Female controls (F_con_)

In total, 81 females (51 patients and 30 controls) were included in the analysis. No differences were found between the two groups in relation to age (P = 0.244) or BMI (P = 0.121). However, significant differences were found in the depression subscale of HADS (HADS-D, P ≤ 0.000), anxiety subscale of HADS (HADS-A, P ≤ 0.000), and in the Pain Catastrophizing Scale (PCS, P ≤ 0.000, Table 1). However, mean scores from both subscales of HADS, showed no scores of 11 or higher, indicating lack of anxiety and/or depression in the study participants.

### Pressure pain thresholds and pain intensities

Lower PPTs were found in Fcmp compared to Fcon in the trapezius and tibialis anterior muscles (P < 0.05, Table 2). One control and four patients were excluded from data analysis due to missing values. Pain intensities were higher in Fcmp at all-time points of evaluation during the MD (P ≤ 0.05, Fig. 1B).

### Interstitial concentration of the metabolites and local blood flow

**Figure 3:**
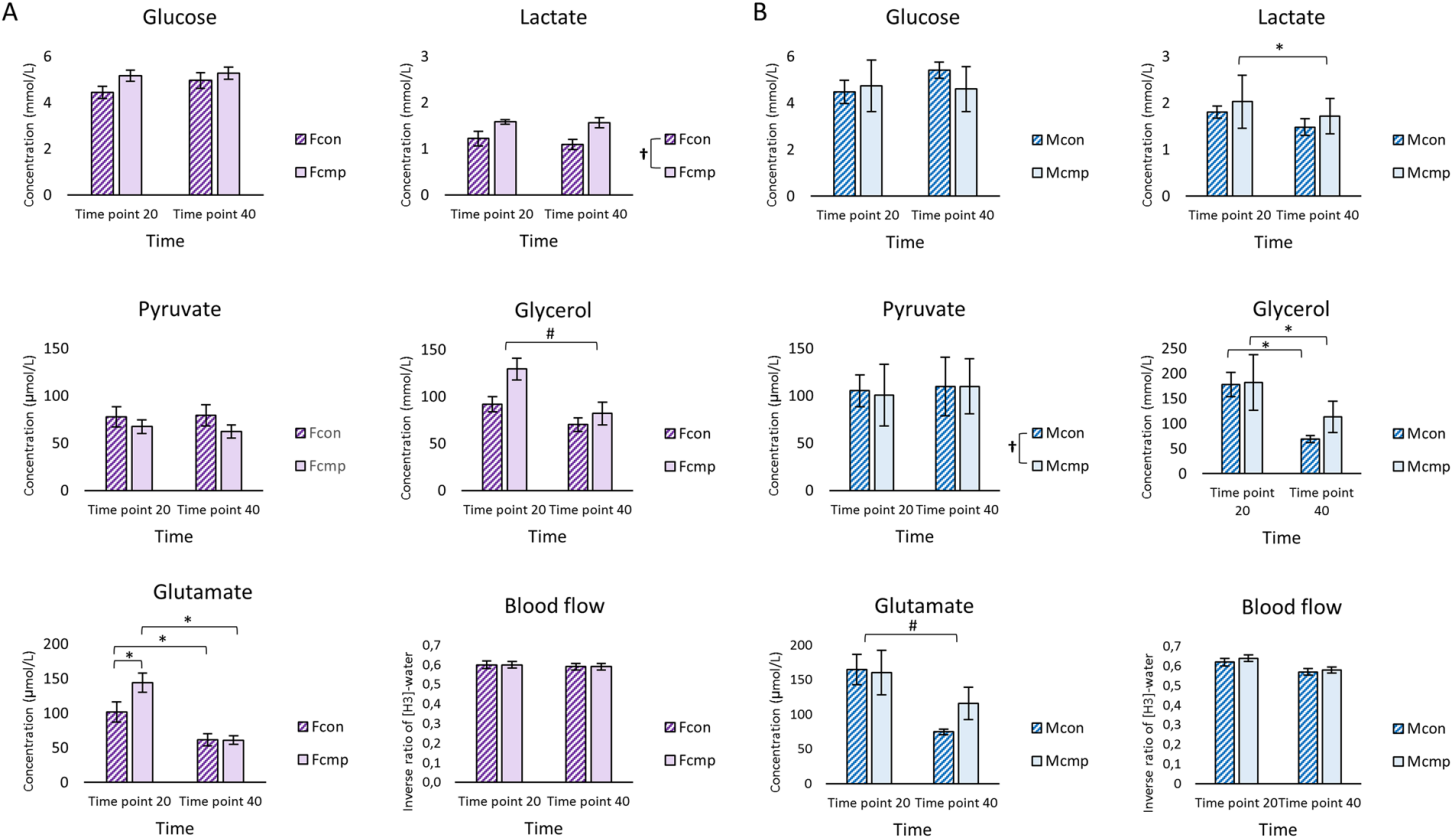
Interstitial concentration (mean and SEM) of muscle metabolites; Glucose, Lactate, Pyruvate, Glycerol, Glutamate and nutritive blood flow (inverse ratio of [^3^H]-water) collected during microdialysis at two time points of 20 min and 40 min in; (**A**) female patients and female healthy controls and (**B**) in male patients and male healthy controls. (*P ≤ 0.05) denotes significant interaction between time*group, (^#^P ≤ 0.05) denotes difference in time. (^†^P ≤ 0.05) denotes significant difference in group. Abbreviations: Fcon = female c ontrols, Fcmp = female patients, Mcon = male controls, Mcmp = male patients Mmol/L = millimol per. Liter, µmol/L = micromol per. Liter.

A significant interaction was found between the female groups and time on the concentration of glutamate (ANOVA: F = 4.884, P = 0.030). A decrease in the concentration of glutamate was found from 20 min to 40 min in both groups; (Fcmp: P = 0.000, Fcon: P = 0.020). However, at 20 min, Fcmp showed higher levels of glutamate (143.60 ± 13.54 µmol/L) compared to the female controls (101.57 ± 14.41 µmol/L, P = 0.046). No difference was found between the groups after 40 min of sampling (P = 0.985). An effect of time was found in the concentration of glycerol (P ≤ 0.000) with a decrease of 34.43 (18.59–50.28) mmol/L after 40 min. At group level, Fcmp had 0.41 (0.12–0.70) mmol/L higher concentration of lactate than the controls (P = 0.006, Fig. 3). No difference in interstitial blood flow was found between the groups and time points (P ≥ 0.05).

### Male patients (M_cmp_) and Male controls (M_con_)

In total, 18 males (8 patients and 10 controls) were included in the analysis. No significant difference in age (P = 0.068) or BMI (P = 0.051) was found between the two groups (Table 1). In addition, Mcmp had significantly higher scores in the pain aspects of HADS-D (P = 0.020) and PCS (P = 0.019) compared to Mcon; although the mean HADS scores in this group were within the normal ranges (e.g. lack of anxiety and/or depression, Table 1).

### Pressure pain thresholds and pain intensities

Mcmp showed lowered PPTs bilaterally over the trapezius muscle (P ≤ 0.05), but no difference was found in the reference muscle between Mcmp and Mcon (P = 0.247, Table 2). Higher pain intensities were found throughout the MD in Mcmp compared to Mcon (P ≤ 0.05, Fig. 1C).

### Interstitial concentration of the metabolites and blood flow

There was an interaction between the male groups and time on the concentration of lactate (ANOVA: F = 5.089, P = 0.038) and glycerol (ANOVA: F = 4.533, P = 0.049) respectively (Fig. 3). A decrease in the concentration of lactate was found in the Mcmp group only (P = 0.032) from 20 min to 40 min. A decrease in the concentration of glycerol was found after 40 min in both groups; (Mcmp: P = 0.005, Fcon: P = 0.021) respectively, but no difference was found between the groups at either time points (20 min: P = 0.135, 40 min: P = 0.211).

A decrease of 74.02 (46.5–101.5) µmol/L in the concentration of glutamate was found after 40 min (P ≤ 0.000). At group level, Mcmp had 32.01 (18.4–45.6) µmol/L lower concentration of pyruvate compared to the male controls. Interstitial blood flow was unchanged between the two male groups (P = 0.697) at both time points (P* = *0.985, Fig. 3).

### Controls (CON), patients with regional pain (Reg), and patients with generalized pain (Gen)

Based on the report from the clinical examination, all participants were further divided into the following subgroups; CON (n = 40), Reg (n = 35) and Gen (n = 24). One control and two patients were excluded from the analysis of the pain questionnaires due to lack of response. A significant difference in weight was found between the two patient groups (P = 0.016) as well as a difference in BMI was found between Con vs. Gen (P = 0.001), and between Reg vs. Gen (P = 0.006) respectively. Furthermore, both subgroups of patients showed higher scores in the pain aspects of HADS-D, HADS-A, and PCS compared to CON (P ≤ 0.05), but no difference was found between Reg and Gen. Gen showed the highest score within both subscales of HADS, however all scores were within normal ranges^15^ (Table 1).

### Pressure pain thresholds

Comparing PPTs over the trapezius and tibialis anterior muscles, lower PPTs were found in Reg and Gen in comparison to CON on both sides of the trapezius muscle and in the reference muscle, tibialis anterior (P  ≤  0.05, Table 2).

### Interstitial concentration of the metabolites and local blood flow

There was an interaction between the groups and time on the concentration of glucose (ANOVA: F = 4.455, P = 0.014), glycerol (ANOVA: F = 4.578, P = 0.013), and Glutamate (ANOVA: F = 4.012, P = 0.022, Table 3). From 20 min to 40 min a significant increase in the concentration of glucose was found in Con (P = 0.046) and Reg (P = 0.035), but not in the Gen (P = 0.120). No differences was seen between the groups at time point 20 min (P = 0.177) or at time point 40 min (P = 0.075). A decrease in the concentration of glycerol was found in all three subgroups from 20 min to 40 min; (Con: P = 0.001, Reg: P = 0.031, Gen: P = 0.001). At time point 20 min, only Gen showed significantly higher levels of glycerol compared to Con (55.76 ± 22.7 µmol/L, P = 0.42). No difference in the concentration of glycerol was found between the groups at time point 40 min (P = 0.264). A decrease in the concentration of glutamate from 20 min to 40 min was found in all three groups; (Con: P = 0.000, Reg: P = 0.000, Gen: P = 0.000). At time point 20 min, Gen showed significantly higher levels of glutamate compared to the Con (78.30 ± 26.4 µmol/L, P = 0.016) and Reg (77.88 ± 26.2 µmol/L, P = 0.016) respectively. No differences was found between the groups after 40 min (P = 0.283). Interstitial blood flow was unchanged between the groups and time points (P ≥ 0.05, Table 3).

**Table 3:** Interstitial metabolite concentration and local blood flow in the trapezius muscle from healthy controls, patients with regional pain and patients with generalized pain.

Time points (min)	CON (n = 40)	Reg (n = 35)	Gen (n = 24 )	Statistic (P-value)
**[Glucose] (mmol/L)**				
20	4.4 (1.5)	5.0 (1.6)	5.2 (2.3)	0.014*
40	5.1 (1.6)	5.6 (1.5)	4.4 (2.4)	
**[Lactate] (mmol/L)**				
20	1.4 (0.8)	1.6 (0.7)	1.7 (1.2)	0.157
40	1.2 (0.6)	1.7 (0.7)	1.4 (0.8)	
**[Pyruvate] (µmol/L)**				
20	84.9 (55.6)	77.9 (53.2)	61.4 (66.2)	0.869
40	87.1 (71.1)	76.1 (57.4)	56.8 (48.9)	
**[Glycerol] (mmol/L)**				
20	114.5 (64.9)	119.9 (81.5)	170.7 (107.9)	0.013*
40	69.8 (34.8)	92.9 (98.4)	74.8 (35.9)	
**[Glutamate] (µmol/L)**				
20	118.2 (78.9)	118.7 (73.4)	200.2 (102.1)	0.022*
40	65.0 (41.7)	60.8 (50.5)	85.8 (46.7)	
**Time points (min)**	**CON (n = 40)**	**Reg (n = 35)**	**Gen (n = 24)**	**Statistic (P-value)**
**Blood Flow (outflow-to-inflow ratio of H**_**2**_ **0** ^**3**^ **)**				
20	0.63 (0.9)	0.63 (0.1)	0.63 (0.7)	>0.05
40	0.63 (0.1)	0.63 (0.1)	0.62 (0.1)	

Collected during 20 and 40 min of MD. Values are presented as mean and standard deviation (SD).*Denotes an interaction between time and group (time*group), analyzed by a two-way mixed model ANOVA. Abbreviations: CON = controls, Reg = patients with regional pain, Gen = patients with generalized pain, Mmol/L = millimol per. Liter, µmol/L = micromol per. Liter, n = number of included subjects.

## Discussion

This study measured the level of different metabolites within trapezius muscle milieu following acute trauma caused by the insertion of a MD probe and additionally, investigated if any changes in response to the tissue trauma from 20 min to 40 min differed between CMP patients and healthy controls. The metabolic response pattern changed over time and alterations in the level of metabolites from 20 min and 40 could be seen in both CMP and healthy controls. Furthermore, pain questionnaires and pain intensities manifested clinical aspects of pain closely to what CMP patients usually describe.

Increased concentrations of pyruvate and lactate have been identified after catheter insertion in an animal model of inflammatory trauma following an experimental tissue trauma^16^. However, no difference was found between the injured muscle and the control muscle. Levels of pyruvate and lactate have been shown altered in different types of chronic pain conditions e.g. increased concentration of pyruvate and lactate have been measured in females with work-related muscle pain and chronic trapezius myalgia^17,18^. However, the metabolite alteration in these pain conditions is often suggested as a result of insufficient oxygen supply to the muscle tissue and thereby a change to anaerobic conditions^19^. But, it is not consistent evidence in existing literature that lack of sufficient blood flow or alteration in the flow would affect tissue’s oxygen supply^17,18,20^; therefore, alterations in tissue blood flow cannot be the only explanation for increased levels of pyruvate and lactate. In our study, however, the MD procedure was performed in a resting muscle with no obvious difference in respect to interstitial muscle blood flow. Increased concentrations of lactate in patients with fibromyalgia have been linked to mitochondrial insufficiency in the trapezius muscle^21^. However, based on a review from 2004 by Gladden^22^, increased levels of lactate is not solely associated to tissue hypoxia but function as important interim in wound restoration and tissue generation. In this study, the concentration of lactate was increased after MD insertion in CMP except for the male subgroup in which the level of lactate decreased in the patients after 40 min. In comparison to the initial trauma study investigated by Gerdle *et al*.^14^ elevated concentrations of lactate in CNSP was found compared to the controls.

Results from different MD studies seem to be in conflict determining glutamate as a biochemical marker for chronic muscle pain. Two studies showed inconsistent findings of glutamate levels in females patients with fibromyalgia^8,10^ whereas other studies provide evidence on increased levels of glutamate in different pain conditions^17,23^. Glutamate is a fundamental excitatory neurotransmitter and its transmission through sodium-dependent glutamate transporters^24,25^ is important in both normal and pathological nociception^26^. Normally, these transporters maintain low levels of extracellular glutamate due to its neurotoxic ability^27^; however, as a consequence of altered long-term depolarization and negative feedback mechanisms, glutamate is believed to induce pain through release from peripheral afferents terminals^28^. Overall, findings from this study shows that the level of glutamate decreases after 40 min of MD insertion in both patients and controls. This may indicate that both patients and healthy controls show a nociceptive response of the tissue due to the trauma in which glutamate is passively released and found in higher levels in the tissue at the beginning of MD sampling. Additionally, in the female subgroup, higher concentration was found in the patients after 20 min sampling compared to the controls but interestingly, no difference was seen after 40 min of sampling.

Glycerol is an important compound in fatty acid metabolism^29^ and synthesized in muscle and fatty tissues for further use as energy substrate^30^. Additionally, high levels of glycerol have been associated to acute brain injury and membrane phospholipid degradation^31^. As glycerol is part of the human cell membrane, higher concentration could be expected in the tissue due to cell damage following MD probe insertion. In all groups, the concentration of glycerol decreased after 40 min of sampling suggesting that the fluctuations in the level of glycerol in both patients and controls is affected by the probe insertion. However, from these findings we cannot conclude whether any differences in response to the probe is different between the patients and controls.

Glucose is essential for the maintenance of human body metabolism and is the main energy resource for brain activity and neuronal networking^32^. As glucose is transported through the blood stream, its concentration may reflect metabolic usage throughout the different body compartments and tissue structures. In this study, only an increase in the level of glucose was found in the subgroup analysis in the CMP population Reg and in the controls. As these findings only apply for a smaller subgroup population we suggest that an acute trauma does not seem to have any significant effect on the glucose concentration in the muscle interstitial space. But rather, it may reflect ordinary alterations occur in the blood flow.

In all group comparisons between patients and controls, significant differences were found regarding the different instruments of the applied questionnaires, values of PPTs, and the pain intensities. In general, all patient groups considered their current situation more negatively in comparison with healthy individuals for all perspectives of physical and emotional aspects of pain. These findings have been confirmed by other pain studies and our findings only support the various aspects to be considered in treatment strategies for pain patients^33,34^. However, as the CMP and controls seem to act differently towards the MD trauma responses, the metabolic pattern may not reflect the clinical situation per se.

The trauma phase is considered to last approximately <2 hours^11,12^. Whether metabolic alterations occur during this acute trauma phase are less understood and the initiative was taken by Gerdle *et al*.^14^. However, this study was the first to investigate the nociceptive reaction of the tissue within the very initial phase of the trauma period. Changes were seen in certain metabolites from 20 min to 40 min, however this sampling period may be too short to reflect any reliable altered metabolic pattern for an acute nociceptive response in CMP. Moreover, as these findings only applied for a smaller subgroup population we consider this study to be a preliminary study and that our results may reflect trends rather than absolute changes to draw any sharp conclusions.

Only few substances have been investigated with MD^35^ and more will be important to include in further studies in order to investigate the effect of an acute trauma in different chronic pain patients. Primarily, only females have been investigated in chronic pain studies, which in turn may reflect the high prevalence of this patient group in CMP^2^. In this study, male participants showed similar response pattern as the female subgroup; however, further studies are required to clarify this finding and additionally, further examine the complexity of sex-related factors and biochemical variations. Several MD studies have previously been performed on small sample sizes making a high risk of inconclusive results and hence, failure to detect any clinically important differences between patient groups^35^. Based on previous findings by Rosendal *et al*.^23^, a minimum cohort of 23 subjects should be included to detect a clinical difference of 1.7 mmol/L in the concentration of lactate between patients and healthy individuals. As this present study is investigating a new aspect on metabolic response patterns, previous finding may not exclusively be applicable for this. However, results need to be confirmed in a larger scale study to prove or disprove the effect of sample size. Furthermore, including diverse groups of CMP might be important to profile metabolic responses to acute phase of MD probe insertion in certain subgroups of CMP. Due to the chosen sampling time of 40 min in the trauma phase, the results should be cautiously interpreted until a larger scale study being conducted.

## Materials and Methods

### Study design

This study was designed as a cross-sectional study investigating different metabolites in microdialysate in response to an acute nociception (probe insertion) in the trapezius muscle in patients with CMP and healthy individuals. The study was approved by The Regional Ethical Committee of Linköping University (project number: 2013/151-31) and was carried out at the PAINOMICS® Laboratory, Rehabilitation Medicine, Department of Medicine and health science, Linköping University, Sweden, within the period of 2014 to 2016. The study consisted of one session lasting approximately 2 hours and was conducted following the GCP guidelines and the declaration of Helsinki was taken into account.

### Subjects

Fifty-nine patients (51 females and 8 male) with chronic musculoskeletal pain (CMP) and mean age (SD) of 43.7 (10.6) years were recruited through review of medical reports of former patients and from the Pain and Rehabilitation Center, University Hospital, Linköping. Forty (30 females and 10 male) healthy and pain free controls (CON) with a mean age (SD) of 39.6 (8.9) were recruited via local advertisement. Both groups of participants were assessed for eligibility through a routine medical examination at the clinic, which included information on weight, height, blood pressure and use of medications. The examination of pain patients also included current pain status, possible diagnosis, palpation of tender points and the localization of the pain marked on a standardized drawing of a body chart. Exclusion criteria in both groups were diabetes mellitus, inflammatory disease, neurological disease, systemic diseases, thyroid disease, heart and/or lung diseases or psychiatric disorders. Prior to the clinical session the participants were instructed not to take any non-steroidal anti-inflammatory drugs (NSAIDs) and to refrain from any strenuous exercise 48 hours before the examination. On the actual test day, the participants were not allowed to drink any caffeine-containing beverage, smoke or use any analgesics before the MD was performed. Additionally, any deviations from the instructions were noted. Upon meeting the eligibility criteria, baseline assessments for pressure pain thresholds (PPTs) over the trapezius muscle and the tibialis anterior muscles were determined for all participants. Subsequently, the MD was conducted in the trapezius muscle on the dominant side (CON) or the most painful side (CMP) of the participants and all subjects rated their pain intensity on a numerical rating scale (NRS, 1–10) before insertion of the MD probe, after 20 and 40 min, and once upon the completion of MD. After examination, the participants were given a set of questionnaires characterizing different aspects of pain. All participants gave their written informed consent before the clinical examination was initiated.

### Questionnaires

The following instruments; Hospital Anxiety and depression Scale (HADS) and Pain Catastrophizing Scale (PCS)^36^, (Swedish validated versions) were included in this study. The HADS is divided into two subcategories including 7 items. Each item is rated on a 4-point scale (0–3) resulting in a sum score ranging from 0–21. A score of 0–7 for either subcategory indicate normal ranges, score of 8–10 indicate suggestive presence, and score above 11 indicate potential presence of anxiety and/or depression. The PCS is rated on a 5-point scale (0 = not at all, 4 = all the time), resulting in a total sum score ranging from 0–52.

### Algometry

Mechanical stimulation of the trapezius and tibialis anterior muscles were performed by a handheld electronic pressure algometer (Somedic, Hörby, Sweden) with a standard flat-ended probe of 10 mm in diameter. Pressure was applied perpendicularly on the skin overlaying the region of interest with an increase rate of 30 kPa/s and a maximum of 600 kPa/s. The participants were instructed to report their PPTs by pressing a stop button when the sensation of the increasing pressure changed to the first sensation of pain. The stimulus intensity was then noted. PPT was determined on both sides of the trapezius muscle and conducted at two time points with a reference measure of PPT obtained over the tibialis anterior muscle on both legs. The average of two readings was calculated and used for further analysis and comparisons.

### Microdialysis

The MD technique in the trapezius muscle was conducted as previously described^8,10,37^. Briefly, prior to insertion of the MD probe, the skin and subcutaneous tissue were anaesthetized with a local injection (0.5 mL) of Xylocaine (20 mL/mg). Two MD catheters (20 kDa cut-off point, membrane 30 mm length, 0.5 mm diameter; CMA Microdialysis AB, Solna, Sweden) were inserted into the upper descending part of the muscle positioned halfway between the lateral border of acromion and pros. spinosus C7 with an inter-catheter distance of 2 cm. To reduce resistance from upper layers, a guided cannula was used to facilitate the entrance of the catheters into the muscle. A high-precision syringe pump (CMA 107; Carnegie Medicine, Solna, Sweden) with a flow rate of 5 µL/min was used to perfuse the catheters with a Ringer acetate solution (Fresenius Kabi AB, Uppsala, Sweden) containing 3 mM glucose and 0.5 mM lactate, 0.3 µL/m [^14^C]-lactate (specific activity 5.77 GBq/mmol; Amersham, Bucks, UK) and 0.3 µl/mL ^3^H_2_O (specific activity 37 MBq/gram). Glucose and lactate were used to mimic the component of biofluid presented in the intramuscular space^38^, and radioactive [^14^C]-lactate and trihydrate were added to calculate relative recovery (RR)^39^ and nutritive muscle blood flow (BF)^40^. After the MD probe insertion, samples were collected every 20 min during a period of 40 min. Each MD vial to collect the obtained samples was weighted on electronic scale before and after the procedure in order to verify consistent sampling to the actual flow rate set. All vials were placed on ice throughout the experiment and subsequently, the dialysate were transferred to Eppendorf tubes and stored at −70 °C until analysis.

### Relative recovery (RR) and nutritive muscle blood flow (BF)

3 mL of scintillation fluid (High-flash Point, Universal LSC-Cocktail, ULTIMA GOLD; PerkinElmer inc.) was mixed with 5 µL of the dialysate or 5 µL of the perfusate into a counting vial and vortexed. Secondly, β-counting was performed by the use of a liquid scintillation counter (Beckman LS 6000TA; Beckman instruments Inc., Fullerton, CA). RR was calculated as the extraction efficiency^41^ and further used to determine the true concentration of each analyte in the extracellular fluid. Nutritive trapezius muscle blood flow (BF) was estimated by the MD ethanol technique, using ^3^H_2_O instead of ethanol^40^ as the ratio of ^3^H_2_O in the dialysate and perfusate (outflow-to-inflow ratio of ^3^H_2_0) inversely depends on the local blood flow in the muscle^42^. RR and BF were calculated as previously described in details by Olausson *et al*.^41^.

### Analysis of metabolites

Dialysates were analyzed for concentrations of glucose (Gluc), lactate (Lac), pyruvate (Pyr), glycerol (Gly) and glutamate (Glu) by the use of ISCUSSflex Analyzer (CMA Microdialysis AB, Solna, Sweden). The MD analyzer measures the concentrations using enzymatic degradation and colorimetric measurements and is calibrated against a standard concentration of each metabolite. Ranges of detection levels were presented as; [Gluc]: 0.1 to 25 mmol/L, [Lac]: 0.1 to 12 mmol/L, [Pyr]: 10 to 1500 µmol/L, [Gly]: 10 to 1500 mmol/L, [Glu]: 1.0 to 150 µmol/L.

### Statistics

The statistical analysis was performed using SPSS (IBM SPSS version 23). Normality was checked by Shapiro-Wilk test and a P ≤ 0.05 was considered statistically significant. Descriptive statistics are presented as mean (SD). To evaluate demographic and clinical data, One-Way ANOVA (two-tailed) were used for group comparisons. The Bonferroni post hoc test was used in cases of significant differences between the groups. For analysis of interstitial concentration of each metabolite and muscle blood flow at the different time points respectively, a repeated analysis of variance (Two-way mixed-model ANOVA) was used with the factors time (20 min and 40 min) and group (patients and controls).
